# Mavacamten facilitates myosin head ON‐to‐OFF transitions and shortens thin filament length in relaxed mouse skeletal muscle

**DOI:** 10.14814/phy2.70606

**Published:** 2025-10-27

**Authors:** Michel N. Kuehn, Nichlas M. Engels, Devin L. Nissen, Johanna K. Freundt, Weikang Ma, Samantha P. Harris, Thomas C. Irving, Wolfgang A. Linke, Anthony L. Hessel

**Affiliations:** ^1^ Institute of Physiology II University of Muenster Muenster Germany; ^2^ Department of Cellular and Molecular Medicine University of Arizona Tucson Arizona USA; ^3^ BioCAT, Department of Biology Illinois Institute of Technology Chicago Illinois USA

**Keywords:** mouse, myosin inhibitors, ultrastructure, X‐ray diffraction

## Abstract

The first‐in‐its‐class cardiac drug mavacamten shifts myosin heads towards a structurally inactive position where they lay along the helical tracks of the thick filament. However, mavacamten is not completely specific to cardiac myosin and can also affect skeletal muscle myosin, an important consideration since mavacamten is administered orally and so will also be present in skeletal tissue. Indeed, emerging clinical reports indicate mavacamten‐induced generalized skeletal myopathy in elderly patients. These findings raise important safety considerations for vulnerable populations, while also highlighting the drug's potential as a novel basic research tool to probe thick filament regulation and myosin head availability in skeletal muscle mechanics experiments. Using small‐angle X‐ray diffraction (MyoXRD), we tracked these structural changes in the thick filaments of relaxed muscle before and after mavacamten incubation and found that mavacamten treatment reduced the proportion of myosin heads in an active state but did not eliminate length‐dependent structural changes in passive muscle that are linked to changes in contraction performance upon activation, demonstrating similar effects to those observed in cardiac muscle. These findings provide valuable insights for the potential use of mavacamten as a tool to study skeletal muscle contraction across striated muscle.

## 1 INTRODUCTION

**FIGURE 1:**
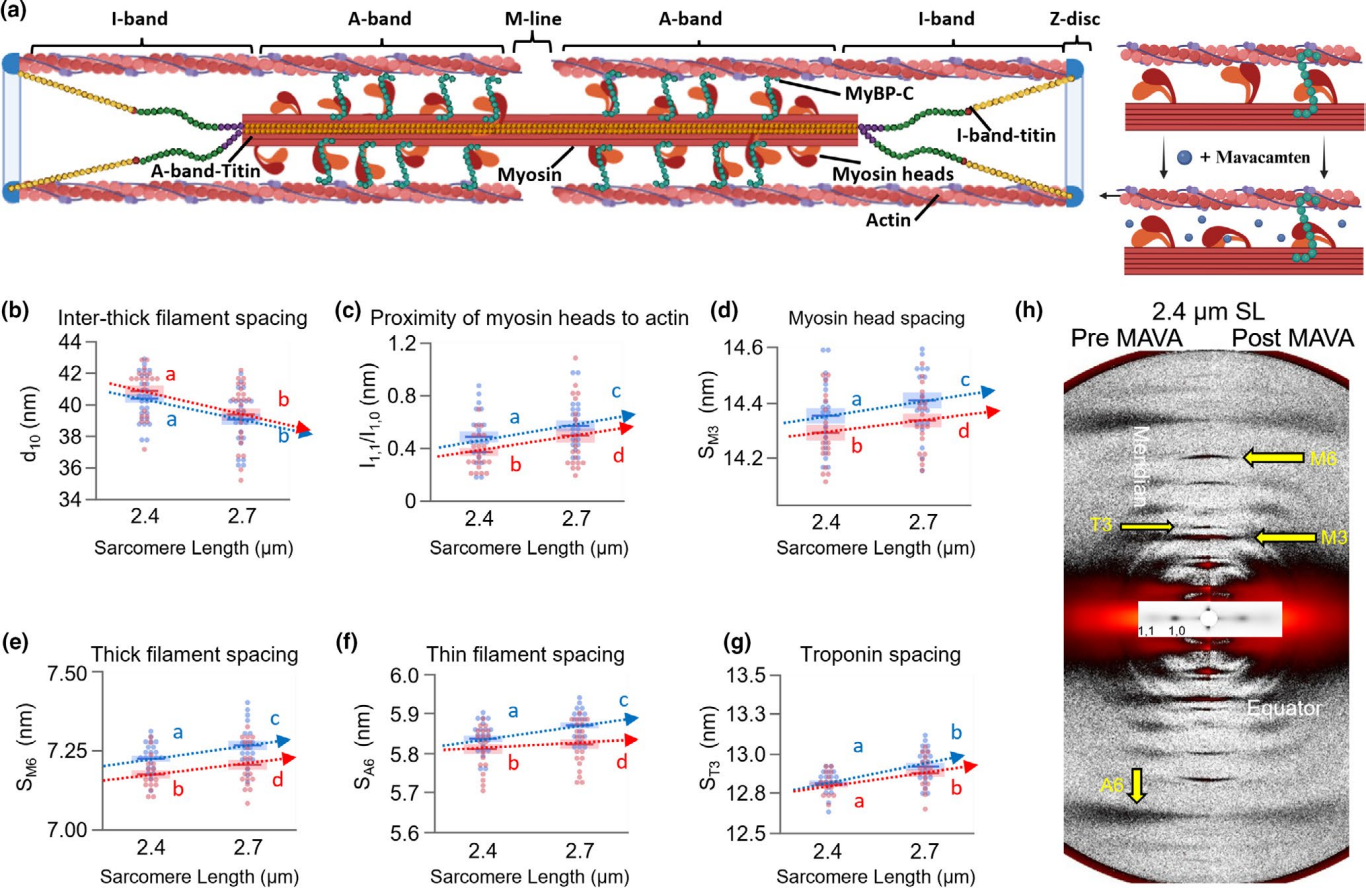
Sarcomeric structures before (blue) and after (red) mavacamten. (a) Cartoon of a passive sarcomere incubated with mavacamten. d_10_ (b), I_1,1/I1,0_ (c), S_M3_ (d), S_M6_ (e), S_A6_ (f) and S_T3_ (g) provided. MyoXRD pattern pre and post mavacamten (MAVA) incubation (h). Data presented as mean ± s.e.m. Statistical significance indicated by connecting letters, where conditions with different letters are significantly different. 2‐way ANOVA with individual random effect and repeated measure design. Further details in Table 1.

In the presence of calcium (pCa < ~7), active tension generation in sarcomeres is produced by crossbridge cycling between the myosin motors in the thick filament and actin in the thin filaments (Figure 1a) (Huxley & Simmons, 1971). The performance of sarcomeres during contraction is partially set in the relaxed condition by setting up the propensity of myosin heads to form crossbridges upon activation. Slight changes to the number of available myosin heads in relaxed sarcomeres have outsized consequences for contraction (Linari et al., 2015; Kampourakis et al., 2016) and create a unique structural fingerprint that is measurable via small‐angle X‐ray diffraction (MyoXRD) (Ma & Irving, 2022). In relaxed sarcomeres (Figure 1a), each of the ~300 myosin heads of a thick filament is in a structural state between an OFF conformational state, where the myosin heads are folded onto their tails and docked into helical tracks along the thick filament backbone, and an ON conformational state, where myosin heads are positioned up and away from the thick filament and towards the thin filament (Farman et al., 2011; Ma & Irving, 2022). As reported previously, stretching a passive muscle fiber from 2.4 to 2.7 μm sarcomere length (SL) increases passive force via stretch of titin string proteins, and is correlated to changes in the sarcomeric structure (Hessel et al., 2022).

SL‐dependent recruitment of myosin heads from the OFF state to the ON state in relaxed muscle has been associated with the length‐dependence of calcium sensitivity (Ma et al., 2021), although the two phenomena are distinct. In this situation, increasing the SL in relaxed sarcomeres increases the number of myosin heads available for cross‐bridge formation upon calcium activation (e.g., OFF to ON transition), thereby enhancing calcium sensitivity and force production (Ma et al., 2021). In support of this notion, mavacamten reduced calcium sensitivity in skeletal muscle (Scellini et al., 2021), and our data support the idea that this could be contributed by mavacamten reducing the number of ON myosin heads in the relaxed sarcomere. Recently, a first‐in‐its‐class cardiac drug was approved to treat certain forms of hypertrophic cardiomyopathies, called mavacamten (marketed as Camzyos) (Walklate et al., 2022), which functions as a myosin motor inhibitor that shifts cardiac myosin motors from the ON towards the OFF state and reduces contraction tension (Kawas et al., 2017; Awinda et al., 2020; Ma et al., 2023). Of relevance for this orally administered drug, mavacamten is not specific to the cardiac myosin isoform and binds skeletal muscle, albeit with reduced effect (Kawas et al., 2017). Indeed, one clinical report has already described a case of generalized skeletal myopathy in an elderly patient treated with mavacamten, raising concerns about its off‐target effects and underscoring the need to better understand its impact on skeletal muscle performance (Amofa et al., 2025). Currently, the MyoXRD‐deduced structural effects of mavacamten and other upcoming small‐molecule therapeutics for cardiac health in skeletal muscle are unknown. However, some mechanical studies indicate that mavacamten reduces active tension in skeletal muscle, albeit with less potency compared to cardiac tissue (Scellini et al., 2021), and is more effective in slow vs. fast skeletal fiber types (Walklate et al., 2022). In addition, mavacamten increases the proportion of inactive myosin heads in both the C‐ and D‐zones of skeletal thick filament filaments by reducing ATP turnover (Pilagov et al., 2023), indicating a broad effect across the thick filament.

Here, we define the myofilament structural signature of resting wildtype (WT) skeletal fibers before and after treatment with mavacamten at a physiological short and long SL. We predicted that mavacamten treatment would produce similar structural effects to those seen in cardiac preparations. We found that mavacamten treatment shifts myofilament structures towards an OFF conformation and alters thin filament structures in a way consistent with those seen in cardiac sarcomeres, demonstrating mavacamten functionality across striated muscles.

## 2 RESULTS

X‐ray diffraction patterns were collected and analyzed from resting (pCa >8) permeabilized fiber bundles at 2.4 and 2.7 μm SL, before and after incubation in mavacamten. Full statistics are in Table 1. Interfilament lattice spacing was quantified as the spacing between the thick filament containing d1, 0 planes of the filament lattice (Figure 1 and Table 1) and is quantified using the 1,0‐reflection located on the equatorial plane of the diffraction pattern (Figure 1h) and decreased from short to long SL, as expected, with no detectable effect of mavacamten (Figure 1b).

**TABLE 1:** Statistical details from experiments shown in Figure 1 (B‐G). N = number of preparations included in the condition. The ANOVA analysis *F*‐statistics (*F*) and *p*‐values (*p*) are provided, as well as a connecting letter report from a Tukey's honest significant difference (HSD) analysis on sarcomere length (SL) before (PRE) and after (POST) mavacamten (MAVA) treatment. Data reported as mean ± s.e.m.

MAVA	SL	*N*	Mean	Std err	ANOVA main effect	*F*	*p*	Connecting letter
d_10_ (nm)								
PRE	2.4	27	40.425	0.279	Treatment	1.61	0.21	a
	2.7	24	39.107	0.348	SL	27.23	< 0.0001*	b
POST	2.4	20	40.911	0.345	Interaction	0.12	0.73	a
	2.7	24	39.408	0.387				b
I_1,1/I1,0_ (nm)								
PRE	2.4	22	0.488	0.041	Treatment	6.09	0.02*	a
	2.7	20	0.573	0.042	SL	6.13	0.02*	c
POST	2.4	21	0.374	0.031	Interaction	0.65	0.42	b
	2.7	21	0.499	0.055				d
S_M3_ (nm)								
PRE	2.4	21	14.354	0.028	Treatment	8.92	0.004*	a
	2.7	21	14.410	0.029	SL	7.68	0.008*	c
POST	2.4	19	14.294	0.028	Interaction	2.36	0.130	b
	2.7	20	14.338	0.025				d
S_M6_ (nm)								
PRE	2.4	22	7.226	0.010	Treatment	11.51	0.001*	a
	2.7	20	7.268	0.015	SL	6.44	0.014*	c
POST	2.4	17	7.176	0.013	Interaction	0.80	0.374	b
	2.7	20	7.207	0.015				d
S_T3_ (nm)								
PRE	2.4	18	12.816	0.018	Treatment	4.70	0.035	a
	2.7	21	12.922	0.023	SL	9.52	0.003*	b
POST	2.4	15	12.807	0.017	Interaction	3.62	0.063	a
	2.7	18	12.881	0.024				b
S_A6_ (nm)								
PRE	2.4	22	5.837	0.008	Treatment	26.51	< 0.0001*	a
	2.7	22	5.872	0.008	SL	9.28	0.003*	c
POST	2.4	20	5.812	0.012	Interaction	1.36	0.248	b
	2.7	22	5.824	0.012				d

* Significant (*p* < 0.05).

Myosin head ON/OFF transitions were assessed using two structural parameters: the equatorial intensity ratio (I_1,1_/I_1,0_; Figure 1c), which generally reflects changes in radial mass distribution within the filament lattice primarily due to myosin heads—but may also be influenced by factors such as sarcomere length and lattice spacing (Malinchik & Yu, 1995), and the spacing of the third‐order meridional reflection (S_M3_; Figure 1d), which reports on the axial periodicity of the thick filament and is associated with myosin head conformation and ordering. While not exclusively specific to head position, increases in both parameters under constant lattice conditions are consistent with transitions towards the myosin ON state. Before and after mavacamten treatment, I_1,1_/I_1,0_ and S_M3_ increased from short to long SL, indicative of the well‐known SL‐dependent transition of myosin heads from the OFF towards the ON state (Ma & Irving, 2022). After mavacamten treatment, I_1,1_/I_1,0_ and S_M3_ decreased across SLs, which suggests an ON‐to‐OFF transition of the myosin heads. Although the structural origin of the M6 reflection (S_M6_; Figure 1e) remains somewhat unclear, it has long been used as a marker that relates to the axial periodicities of structures within the thick filament backbone and is commonly used as a proxy for thick filament extension (Ma et al., 2018; Koubassova et al., 2025). Moreover, S_M6_ serves as one of the indicators of thick filament activation where longer S_M6_ is associated with myosin heads transitioning to the ON state (Ma et al., 2023). S_M6_ increased from short to long SL before and after mavacamten treatment; however, mavacamten treatment reduced S_M6_ across SLs (Figure 1e and Table 1). Thin filament length was quantified via the spacing of the A6 reflection (S_A6_; Figure 1f), which represents the periodicity from the pitch of the left‐handed axial turn of the actin double helix (Egelman et al., 1982; Ma & Irving, 2022). Before and after mavacamten treatment, S_A6_ increased from short to long SL (Figure 1f and Table 1). However, mavacamten treatment reduced the S_A6_ across SLs, while not changing the relative magnitude of thin filament elongation from the short to long SL (ANCOVA interaction *p* = 0.12). Finally, we obtained the periodicity of the troponin structure by the spacing of the T3 reflection (S_T3_; Figure 1g). S_T3_ increased with stretch, but mavacamten treatment had no significant effect (Figure 1g and Table 1).

## 3 DISCUSSION

We report that in skeletal muscle, mavacamten treatment leads to a change in X‐ray diffraction signatures that are consistent with a transition of a proportion of myosin heads from the ON to the OFF state, similar to that seen in cardiac muscle (Ma et al., 2021). In mammals, the control of myosin head configuration is typically regulated by both SL‐dependent and SL‐independent mechanisms, which are structurally evaluated here by changes in myosin‐head associated (e.g. S_M3_ and I_1,1/I1,0_) and thick‐filament associated (e.g., S_M6_) X‐ray reflections. Physiologically, these control mechanisms are regulated by sarcomeric proteins such as titin and MyBP‐C (Irving et al., 2011; Li et al., 2019). In our study, we overrode these control schemes with mavacamten, which directly targets the myosin motors and drives them towards an OFF conformation (Kawas et al., 2017; Gollapudi et al., 2021). This led to a SL‐independent decrease in S_M3_, I_1,1/I1,0_, and S_M6_, which is a signature of an ON‐to‐OFF transition of myosin heads (Ma et al., 2021).

SL‐dependent recruitment of myosin heads from the OFF state to the ON state in relaxed muscle has been tightly associated with force production upon activation, especially length‐dependence of calcium sensitivity (Ait‐Mou et al., 2016). In theory, increasing the SL in relaxed sarcomeres increases the number of myosin heads available for cross‐bridge formation upon calcium activation (e.g., OFF to ON transition), thereby enhancing calcium sensitivity and force production (Farman et al., 2011). Notably, mavacamten does affect calcium sensitivity in skeletal muscle (Scellini et al., 2021), and our data support that at least part of this mechanism can be due to the increase of OFF myosin heads in the relaxed sarcomere.

As sarcomeres stretch, myosin head OFF‐to‐ON transition increases force production upon activation, likely regulated by strain generated in the thick filament backbone by the titin I‐band spring pulling on the thick filament (Hessel et al., 2022). We observed OFF‐to‐ON length‐dependent transitions of myosin heads both with and without mavacamten treatment. These observations suggest that the intrinsic mechanisms governing length‐dependent activation are intact even with the pharmacological suppression of myosin activity and align with studies in cardiac tissue (Anderson et al., 2018; Ma et al., 2024). Of note, compared to the short SL, at long SL we identified a positive relationship between increasing myosins in the ON state and increasing thin filament (Anderson et al., 2018) length (inferred from S_A6_), and this relationship was upheld after mavacamten treatment. In cardiac and skeletal muscle, thin filament proteins can be altered by changing the myosin ON/OFF state balance, suggesting myosin head interactions with actin (Ait‐Mou et al., 2016; Hessel et al., 2022).

Assessment of passive cardiac muscle has demonstrated that tension due to crossbridges is present, even if only a few crossbridges are forming (Selby et al., 2011; Donaldson et al., 2012).

In active muscle experiments, small but functionally significant changes in S_A6_ of about 0.4% have been reported due to cross‐bridge forces during contraction and associated thin filament strain (Kiss et al., 2018). In our study, despite the muscle being relaxed and thus cross‐bridge formation limited, mavacamten treatment led to a decrease in S_A6_ of 0.43% and 0.82% at 2.4 and 2.7 μm SL, respectively. This finding suggests that the thin filaments are under strain prior to mavacamten treatment, possibly because of the formation of small numbers of cross‐bridges known to exist under relaxed conditions. It is also possible that the changes in S_A6_ are associated with other changes to protein orientation along the thin filament that change their respective periodicity but do not necessarily lead to a change in filament strain. In this case, the mechanism could involve, in principle, any thick‐thin filament connection such as myosin heads or myosin‐binding protein C (MyBP‐C). This hypothesis could explain a decrease in S_A6_ with mavacamten treatment because the myosin heads shift towards the thick filament backbone, which will both reduce cross‐bridge formation and potentially disrupt the action of MyBP‐C, as they are linked (Hessel et al., 2024). While cross‐bridges remain the most plausible conduit for this coupling, further investigations are needed to confirm the pathway and functional relevance of any such communication in relaxed muscle, possibly by studying intact muscle preparations. Temperature is known to influence the efficacy of mavacamten, with Walklate et al. (2022) demonstrating greater stabilization of the super‐relaxed (SRX) state at 5°C compared to 30°C. Ma et al. (2021) similarly showed that mavacamten alters thick filament structure and ON/OFF transitions in permeabilized porcine cardiac fibers at 22°C–23°C. Our experiments were performed at 27 °C, which falls between these ranges and is within the range used in prior structural studies of cardiac muscle (e.g., Anderson et al., 2018; Ma et al., 2021). Future experiments spanning a broader temperature range will be important to determine how mavacamten's effects vary with temperature, particularly in skeletal muscle.

## 4 LIMITATIONS

This study was conducted using permeabilized, passive murine skeletal muscle fibers to allow controlled and rapid mavacamten treatment. However, this preparation comes with certain limitations to the resolvable MyoXRD features. As previously reported (Hessel et al., 2022, 2024), myosin layer lines 1 (MLL1) and 4 (MLL4), which are typically used to assess the myosin head OFF‐state in intact muscle preparations (Reconditi, 2006), were not well resolvable and so not reported. This restricts our ability to evaluate all thick filament markers of myosin head order and activation state through these established markers. However, collected parameters, such as I_11_/I_10_, provide important details about myosin head orientation, and so we focus on this aspect here. Additionally, the intensity of the M_3_ reflection (I_M3_) was recorded; however, due to technical limitations, we were unable to normalize it between samples, and therefore, we omitted it. Despite these constraints, the current dataset provides robust insights into the structural features of the OFF state, as supported by previous findings (Ait‐Mou et al., 2016; Ma & Irving, 2022). The interfilament spacing in permeabilized muscle fibers is somewhat larger compared to intact. Dextran is often used to compress the lattice back but was not used in our protocol. While this may affect absolute structural parameters (Caremani et al., 2021), the directionality of treatment is maintained. Thus, our pre–post experimental design allows for reliable detection of the relative directionality of changes (Hessel et al., 2022, 2024). A long‐term objective is to conduct these types of experiments in intact preparations. Passive force measurements during MyoXRD experiments were noisy due to stage movement, and so are not reported here, but others have found no impact of mavacamten on passive tension in skeletal fibers (Scellini et al., 2021). While this limits direct correlation between structural changes and passive tension, the SL change from 2.4 to 2.7 μm is modest, and passive forces in this range are typically low. Thus, it seems unlikely that passive tension alone accounts for the observed changes. A force‐titration of mavacamten was not performed in this study, which limits our ability to define its dose–response profile in skeletal muscle and to directly compare it with cardiac muscle, where sub‐micromolar concentrations are sufficient to suppress force generation (Awinda et al., 2020). In contrast, skeletal muscle requires considerably higher concentrations, such as the 50 μM used here, to achieve comparable effects, as previously shown in skeletal myofibrils (Scellini et al., 2021). Future experiments involving force titration across a range of mavacamten concentrations will be important to define the dose–response relationship in skeletal muscle and to compare pharmacological sensitivity with that observed in cardiac muscle.

## 5 MATERIALS AND METHODS

Animal procedures were approved by LANUV NRW (81–02.04.2019.A472). WT mice (age range 4–8 months old, *N* = 8 mice [3 male/5 female]) were bred and housed at the animal care facility of the University Hospital Muenster. Animals were housed with littermates (12:12 light:dark cycle) with standard chow and water ad libitum (Altromin 1324 E EST; Altromin GmbH, Lage, Germany). Following humane euthanasia via cervical dislocation, the psoas muscle was promptly extracted for long‐term storage and subsequently permeabilized (“skinned”) at −20°C via standard glycerol techniques (Hessel et al., 2022).

Storage and permeabilizing solution 1:1 rigor: glycerol; rigor solution contained mM concentrations of KCl (100), MgCl_2_ (2), ethyleneglycol‐bis (β‐aminoethyl)‐N,N,N′,N′‐tetraacetic acid (EGTA, 5), Tris (10), dideoxythymidin (DTT, 1), and protease inhibitors (Complete, Roche Diagnostics, Mannheim, Germany, pH 7.0; Art.‐Nr.: 04693116001). Relaxing solution (composition in mM: potassium propionate (45.3), N,N‐Bis (2‐hydroxyethyl)‐2‐aminoethanesulfonic acid (BES) (40); EGTA (10), MgCl2 (6.3), Na‐ATP (6.1), DTT (10), protease inhibitors, pH 7.0). The samples were then shipped to the BioCAT facility, Advanced Photon Source, Argonne National Laboratories, where all subsequent experiments were conducted. The samples were stored at −20°C until used. On the day of the experiments, samples were extracted from the storage solution and thoroughly washed in relaxing solution.

Fiber bundles (15–30 fibers, 3–6 mm long, *n* = 28 fiber bundles) were prepared and attached to the experimental rig, as described previously (Hessel et al., 2022), and placed them into an experimental apparatus in the MyoXRD instrument at the BioCAT beamline 18D Advanced Photon Source; Argonne National Laboratory, USA; (Ma & Irving, 2019).

To mitigate MyoXRD‐associated sample damage, no sample segment underwent more than one exposure. While measuring structural changes, the experimental approach captured X‐ray images in passive fiber bundles at two SLs across the in vivo physiological operating range. Fiber bundles were consistently exposed to sarcomere lengths in a fixed order, beginning at 2.4 μm SL and then passively stretched to 2.7 μm SL at a rate of 0.1 μm SL/s, followed by a 90‐second hold.

Then, fibers were incubated at 2.4 μm SL in a solution containing 50 μM mavacamten (MedChemExpress; Art. ‐Nr.: HY‐109037R) prepared in relaxing solution for 20 minutes, followed by the same ramp stretch protocol. 50 μM mavacamten was chosen as it produces near‐maximal inhibition of force generation during full activation, as demonstrated in rabbit psoas myofibrils (Scellini et al., 2021). Sarcomere length (SL) was determined by laser diffraction using a 4‐mW Helium–Neon laser. Fibers were set to an SL of 2.4 μm, with a measurement variability of approximately ±0.05 μm, as estimated from the width of the first‐order diffraction peak.

MyoXRD patterns were collected for both conditions (pre and post) at 2.4 to 2.7 μm SL. Experiments were conducted at a temperature of 27°C. Appropriate Box‐Cox transformations were applied to the data to align with normality and homoscedasticity assumptions. Significant main effects were subjected to Tukey's highly significant difference (HSD) multiple comparison procedures. X‐ray diffraction patterns were analyzed using the MuscleX open‐source data reduction package (Jiratrakanvong et al., 2024) and calibrated using the 100‐diffraction ring of silver behenate at d001 = 5.8380 nm. As a quality control measure, data from gaussian fit errors >10% were discarded. Statistical analyses were performed employing JMP Pro (V16, SAS Institute Inc., Cary, NC, USA), and a significance level of *α* = 0.05 was set. The response variables encompassed all relevant X‐ray parameters. An initial repeated‐measures analysis of variance (ANOVA) design was established. In the context of the mavacamten experiments, the model incorporated fixed effects such as treatment (pre/post mavacamten incubation) and condition (2.4 μm SL and 2.7 μm SL), along with a treatment × condition interaction term. Furthermore, the model accounted for a random (repeated measures) effect attributable to individual variability.

## AUTHOR CONTRIBUTIONS

M.N.K. and A.L.H. conceptualized the project; M.N.K., A.L.H., J.K.F., W.A.L., T.C.I. and W.M. developed the methods; M.N.K., A.L.H., W.M., N.M.E. and D.L.N. conducted the investigation; M.N.K. analyzed and visualized the datasets; A.L.H. supervised the study; M.N.K. wrote the original draft; all authors review, edited, and agreed to the final draft; S.P.H. contributed to data and funding acquisition.

## ETHICS STATEMENT

TI provides consulting and collaborative research studies to Edgewise Therapeutics Inc. A.L.H. and M.N.K. are owners of Accelerated Muscle Biotechnologies Consultants LLC. All other authors declare no competing interests.

## Supporting information


Data S1.

## Data Availability

All data used to generate the figures is available in the supplemental information.
